# Reduced cortical neuron number and neuron density in schizophrenia with focus on area 24: a post-mortem case–control study

**DOI:** 10.1007/s00406-022-01513-6

**Published:** 2022-11-09

**Authors:** Richard Gaus, Melanie Popal, Helmut Heinsen, Andrea Schmitt, Peter Falkai, Patrick R. Hof, Christoph Schmitz, Alisa Vollhardt

**Affiliations:** 1 Department of Neuroanatomy, Institute of Anatomy, Faculty of Medicine, LMU Munich, Pettenkoferstr. 11, 80336 Munich, Germany; 2 Morphological Brain Research Unit, Department of Psychiatry, University of Würzburg, Würzburg, Germany; 3 Department of Psychiatry and Psychotherapy, University Hospital, LMU Munich, Munich, Germany; 4 Laboratory of Neuroscience (LIM27), Institute of Psychiatry, University of São Paulo, São Paulo, Brazil; 5 Nash Family Department of Neuroscience and Friedman Brain Institute, Icahn School of Medicine at Mount Sinai, New York, NY USA

**Keywords:** Schizophrenia, Anterior cingulate cortex, Area 24, Von Economo neuron, Stereology, Layer V

## Abstract

Structural and functional abnormalities of the anterior cingulate cortex (ACC) have frequently been identified in schizophrenia. Alterations of von Economo neurons (VENs), a class of specialized projection neurons, have been found in different neuropsychiatric disorders and are also suspected in schizophrenia. To date, however, no definitive conclusions can be drawn about quantitative histologic changes in the ACC in schizophrenia because of a lack of rigorous, design-based stereologic studies. In the present study, the volume, total neuron number and total number of VENs in layer V of area 24 were determined in both hemispheres of postmortem brains from 12 male patients with schizophrenia and 11 age-matched male controls. To distinguish global from local effects, volume and total neuron number were also determined in the whole area 24 and whole cortical gray matter (CGM). Measurements were adjusted for hemisphere, age, postmortem interval and fixation time using an ANCOVA model. Compared to controls, patients with schizophrenia showed alterations, with lower mean total neuron number in CGM (− 14.9%, *P* = 0.007) and in layer V of area 24 (− 21.1%, *P* = 0.002), and lower mean total number of VENs (− 28.3%, *P* = 0.027). These data provide evidence for ACC involvement in the pathophysiology of schizophrenia, and complement neuroimaging findings of impaired ACC connectivity in schizophrenia. Furthermore, these results support the hypothesis that the clinical presentation of schizophrenia, particularly deficits in social cognition, is associated with pathology of VENs.

**Supplementary Information:**

The online version contains supplementary material available at 10.1007/s00406-022-01513-6.

## Introduction

Schizophrenia is a severe neuropsychiatric disorder with serious psychosocial consequences for patients and a considerable public health burden [1]. Despite continuous research efforts since its initial description [2], the pathophysiology of schizophrenia has remained poorly understood [3]. However, reliable findings of structural brain alterations at the macroscopic level [4–10] strongly suggest that schizophrenia has neuropathologic correlates and cannot be understood as a purely functional mental illness, without structural brain involvement [11].

A particular focus of schizophrenia research has been the anterior cingulate cortex (ACC). Consistent neuroimaging and neuropathologic evidence of abnormalities in this region suggests its likely role as a contributor to the pathophysiology of schizophrenia [12]. Key findings include lower mean ACC gray matter volume [6, 8, 9, 12, 13] and lower mean synaptic density [12, 14–16] in the ACC in patients with schizophrenia compared to controls. Although less unanimous, findings of perturbed ACC connectivity have been frequently reported [17–19]. Area 24 is the major subregion of the ACC [20] and it has been thoroughly investigated in a series of studies focusing on the neuropathology of schizophrenia [21–28] as a proxy for the ACC. Focusing on layer-specific alterations in area 24, studies reported not only increased [29] but also decreased [28] and unchanged neuron density [23, 25, 26, 30, 31]. Unlike observations of neuron density, investigations on neuron number in area 24 resulted in no differences [22, 32]. However, investigations of layer-specific alterations of total neuron numbers in schizophrenia using rigorous, design-based stereologic methods have not been reported so far. A striking characteristic of area 24 is the presence in its layer V of so-called spindle or von Economo neurons (VEN), an unusual class of neurons that in humans appear only in a few cortical regions, mostly the ACC and frontoinsular cortex [33]. VENs are likely specialized projection neurons [34–36] and occur in humans, great apes [33, 37] and several other mammalian species [38]. A growing interest in the role of VENs in neuropsychiatric disorders has led to findings of VEN alterations in a number of illnesses [36, 39–41], including schizophrenia [21, 42, 43] for review, see [44]. So far, however, only alterations in the density of VENs in area 24 of patients with schizophrenia were investigated, that revealed no statistically significant differences between patients with schizophrenia and controls [21, 43]. However, lack of alterations of mean cell densities does not predict or imply lack of alterations of total cell numbers [45].

In the present study, we determined total number and density of VENs in layer V in area 24 in postmortem brains (both cerebral hemispheres) of patients with schizophrenia and matched controls using a rigorous, design-based stereologic approach. In addition, we determined volumes of layer V in area 24, whole area 24 and the whole cortical grey matter (CGM), as well as total neuron numbers and neuron densities in these regions of interest (ROIs). Using this approach, it was possible to assess whether an observed alteration in the brains of patients with schizophrenia was specific to the investigated ROI or more general in nature. The investigated brains were the same that were already analyzed in previous studies by our group [31, 46–48], enabling us to interpret the present results in conjunction with previous findings from the same brain specimens.

## Methods and materials

### Brain specimens

This study was conducted on both cerebral hemispheres of postmortem brains from 12 male patients with schizophrenia (aged 50.5 ± 3.4 years [mean ± standard error of the mean, SEM]; postmortem interval 38.1 ± 7.7 h; fixation time 199 ± 25.2 days) and 11 age-matched male controls (aged 54. 5 ± 2.5 years; postmortem interval 23.6 ± 4.3 h; fixation time 1028 ± 432 days). Age at disease onset was 22.6 ± 1.6 years for patients with schizophrenia. Clinical characteristics are listed in Table 1. The brain specimens analyzed in this study are the same that were investigated in earlier studies by our group [31, 46–48]. Brains were collected by H. H. between 1988 and 1994.

**Table 1:** Clinical characteristics of all included subjects

No^a^	A	O	Cause of death	PMI	Fix	Diagnosis	
	[y]	[y]		[h]	[d]	DSM-IV	ICD-10
S1	22	19	Suicide	88	130	295.30	F20.00
S2	36	28	Suicide	< 72	115	295.30	F20.00
S3	46	24	Systemic hypothermia	< 24	327	295.30	F20.01
S4	50	17	Peritonitis	< 24	203	295.30	F20.00
S5	50	22	Suicide	18	170	295.30	F20.00
S6	51	17	Septicemia	33	127	295.60	F20.50
S7	54	20	Septicemia	27	250	295.60	F20.50
S8	55	22	Right-sided heart failure	25	84	295.30	F20.00
S9	57	37	Septicemia	76	163	295.30	F20.00
S10	60	24	Pulmonary embolism	< 48	311	295.30	F20.01
S11^b^	62	19	Aspiration	7	171	295.30	F20.00
S12	63	22	Acute myocardial infarct	15	338	295.60	F20.50
C2	36	–	Gunshot	24	143	–	–
C3	47	–	Acute myocardial infarct	< 24	133	–	–
C5	50	–	Avalanche accident	23	498	–	–
C6	51	–	Septicemia	7	285	–	–
C7	54	–	Acute myocardial infarct	18	168	–	–
C8	56	–	Acute myocardial infarct	60	3570	–	–
C9	58	–	Acute myocardial infarct	28	126	–	–
C10	60	–	Gastrointestinal hemorrhage	18	101	–	–
C11^b^	60	–	Gastrointestinal hemorrhage	27	302	–	–
C12	62	–	Acute myocardial infarct	< 24	3696	–	–
C13	65	–	Bronchopneumonia	6	2289	–	–

*S* patient with schizophrenia, *C* control, *A* age at death, *O* age at onset, *PMI* postmortem interval (time between death and autopsy), *Fix* fixation time, *DSM-IV* Diagnostic Statistical Manual (4th revision) [49], *ICD-10* International Statistical Classification of Diseases and Related Health Problems (10th revision) [50], *y* years, *h* hours, *d* day

^a^These numbers refer to the numbers also used in earlier studies of the same sample by our group [46–48]. Unfortunately, original cases S13, C1 and C4 could not be investigated in this study because sections of these brains were unfortunately damaged. Accordingly, these cases are omitted from the table

^b^The volume, total neuron number and neuron density of the cortical gray matter of these cases could not be analyzed because sections from the frontal and occipital poles were missing

All subjects had been treated in German hospitals. Full medical records were available for the patients with schizophrenia. In addition, autopsy records (including a brief medical history) were available for all subjects. Ethnic backgrounds between patients with schizophrenia and controls were similar but the groups were not fully matched with respect to socioeconomic status and education. All patients with schizophrenia fulfilled the diagnostic criteria of the Diagnostic Statistical Manual (4th revision, DSM-IV) [49] and the International Statistical Classification of Diseases and Related Health Problems (10th revision, ICD-10) [50]. Two experienced psychiatrists assessed the reports to ensure the absence of psychiatric diagnoses in the controls and to verify that the diagnoses of schizophrenia complied with DSM-IV criteria. Subjects with any of the following characteristics were excluded from this study: neurological problems requiring intervention and/or interfering with cognitive assessment, history of recurrent seizure disorder, history of severe head injury with loss of consciousness, history of self-administered intoxication and diabetes mellitus with free plasma glucose > 200 mg/dl.

All patients with schizophrenia were treated with antipsychotics. However, lifetime medication exposures were not available. Autopsies of the subjects were performed after obtaining consent by a relative as required by German law. Tissue extraction and subsequent processing was performed by H. H. or pathologists instructed by him for identical specimen handling and processing procedures. The use of the autopsied subjects for scientific research as described in this study was approved by the responsible institutional review boards.

All brains of patients with schizophrenia and controls older than 40 years were tested for absence of neurofibrillary tangles exceeding Braak’s stage I [51]. This was confirmed on sections through the central portion of the entorhinal and transentorhinal cortex that were not stained with gallocyanin but processed with the Gallyas method [52].

### Tissue processing

**Fig. 1:**
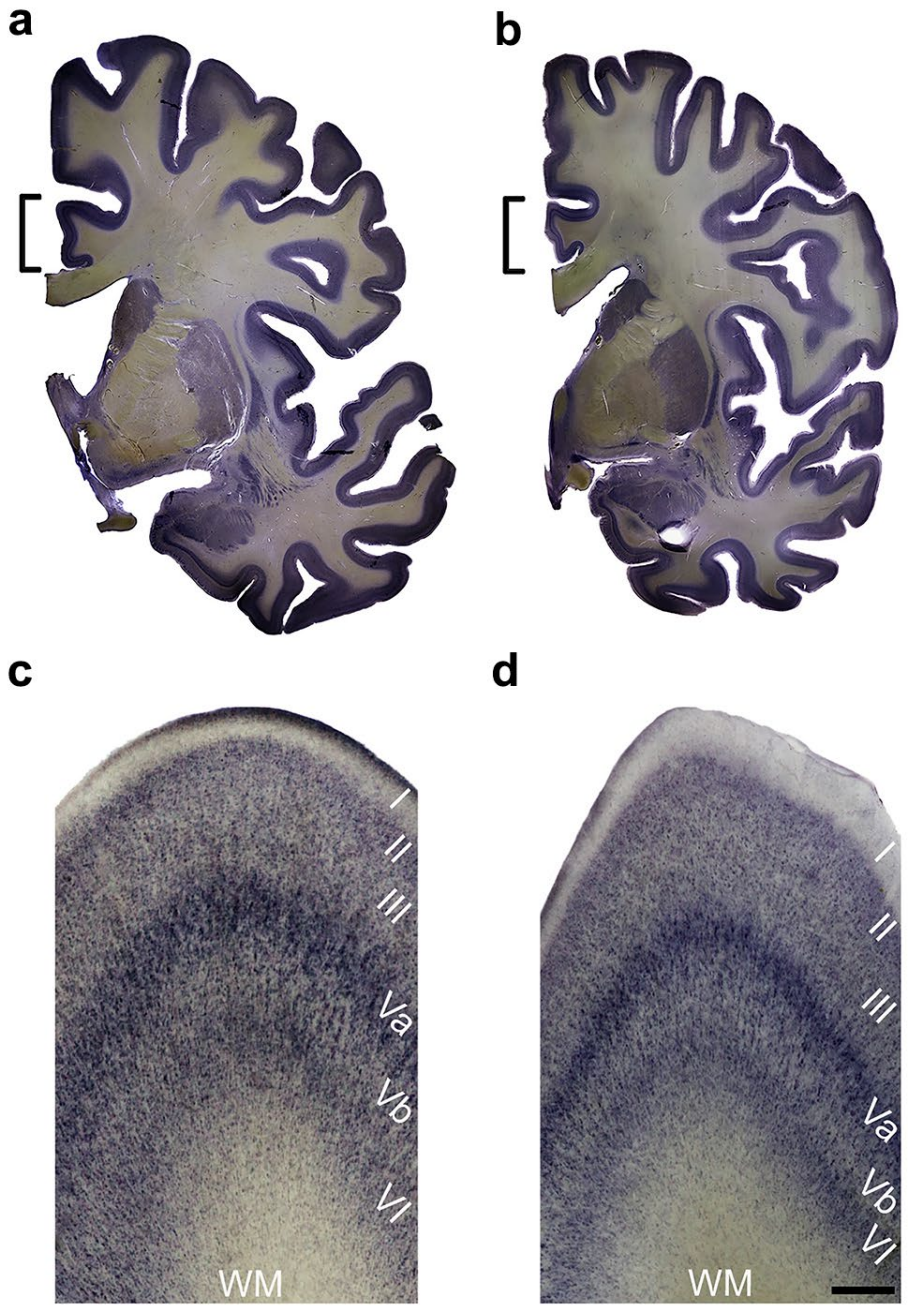
Representative coronal sections of brain hemispheres investigated in this study. Hemispheres from a control (**a**, **c**) and a patient with schizophrenia (**b**, **d**). The brackets in the overview photographs (**a**, **b**) denote area 24, a part of which is each shown in the close-up photographs (**c**, **d**). Layers are indicated in Roman numerals. Scale bar = 10 mm in **a** and **b**, 370 µm in **c** and **d**

The brains were fixed by immersion in 10% formalin (one part 40% aqueous formaldehyde and nine parts water). Following fixation, cerebellum and brainstem were separated from the brain at the rostral end of the pons, after which the hemispheres were divided mediosagittally and the meninges and calcified pial vessels were removed. Then, the hemispheres were pretreated with cryoprotective media, using formaldehyde, dimethylsulfoxide (DMSO) and glycerol as substrates, and subsequently embedded in 3% agarose or 15% gelatin [53]. Next, the tissue blocks were frozen in − 60 °C isopentane. Finally, the tissue blocks were serially cut into 700-µm-thick sections with a freezing microtome (Tetrander, Jung, Nussloch, Germany). Every second or third section was stained with gallocyanin [53] and mounted on microscopic slides (Fig. 1). The remaining sections of the hemispheres were stored in 4% formalin in plastic boxes. All sections were visually examined for the absence of macroscopic and histopathologic alterations, including tumors, infarcts, heterotopias, signs of autolysis, staining artifacts and gliosis.

In one case, the hemispheres of control subject C7 were embedded in celloidin [54] and serially cut into 440–500-µm-thick sections using a sliding microtome (Polycut, Cambridge Instruments, UK). Those slices were also stained with gallocyanin [53].

### Stereological analysis

Three investigators (R.G., M.P. and A.V.) performed the stereologic analyses on both hemispheres of all brains. All investigators were blinded to any clinical characteristics of the subjects, including diagnosis. Analyses were performed on fully equipped stereology workstations (see Supplementary Table 2 for further details on the stereology workstations). Stereologic analysis was performed between 2019 and 2021.

One investigator performed all analyses of the CGM, the second investigator all analyses of area 24 except for the VENs and the third investigator the analysis of the VENs. In addition, the third investigator performed all delineations of ROIs on sections containing area 24. This procedure excluded the possibility that differences in results between patients with schizophrenia and controls could have been introduced by different investigators.

**Fig. 2:**
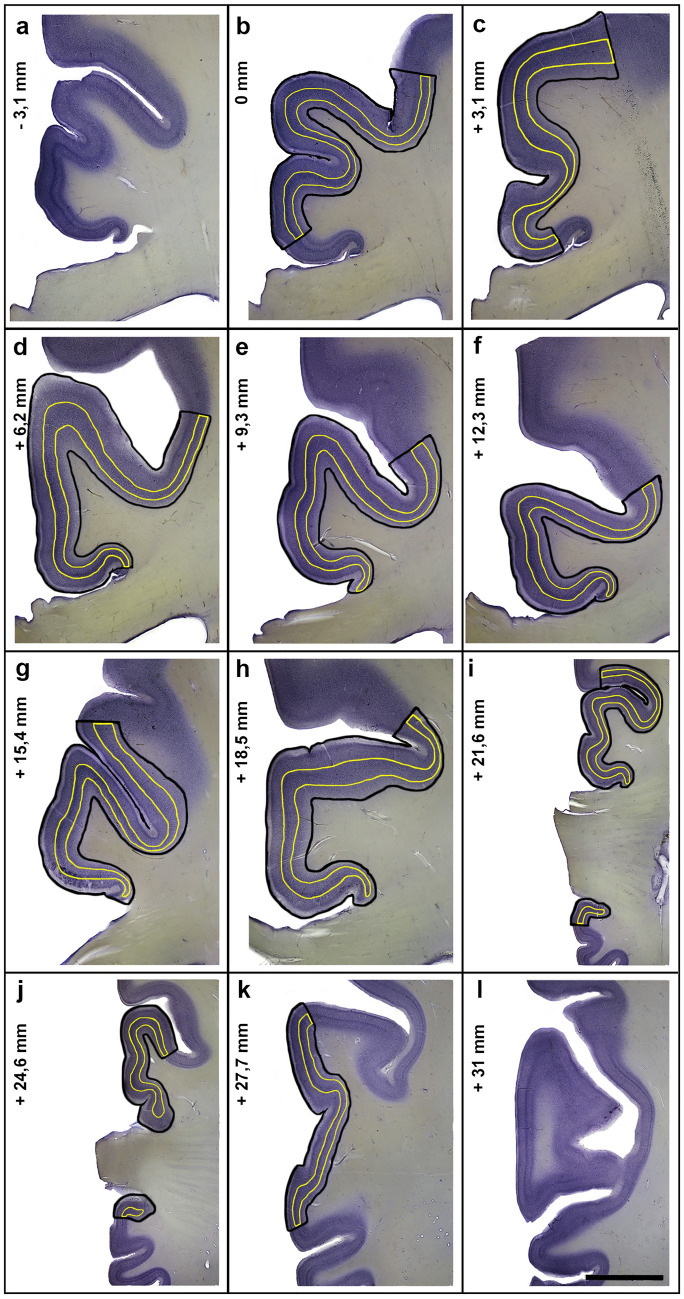
Representative series of coronal sections throughout area 24 in a patient with schizophrenia. Delineations of area 24 (black lines) and its layer V (yellow lines) are indicated. Panels **a** and **l** show the sections caudal and rostral to area 24. The position of the most caudal section showing area 24 (**b**) was defined as 0 mm; the distance between this section and the other sections is indicated in Panels **a** and **c**–**l**. Area 33 and 32 are not indicated. The apparent absence of area 33 is an artifact caused by different shrinkage factors of grey and white matter [100]. Tears were regularly observed (very noticeable on **a** and **g**) at the point of the ventral cingulate gyrus where both, myelin rich corpus callosum and gray matter of the cingulate gyrus are closely attached to each other. Scale Bar = 5 mm in **a**–**h**, 9.5 mm in **i** and **j**, 7.3 mm in **k** and **l**

Three ROIs were delineated at low magnification using a 1.25 × objective: whole CGM, whole area 24 and layer V in area 24. Delineation of area 24 in the ACC was performed according to the cytoarchitectural characteristics described by Vogt et al. [20]. Photographs of the ACC were taken to determine the rostral and caudal borders of area 24 in the hemispheres (1176 photographs in total) (Fig. 2). Simultaneously, the absence of VENs was carefully checked as the presence of these cells is limited to area 24 in the ACC and is therefore a reliable marker for this area [36].

On average, every 9th section showing the CGM and every 5th section showing area 24 (every 6th section in the analysis of VENs) was analyzed, resulting in a systematically and randomly sampled (SRS) series of sections encompassing each ROI. Using the Cavalieri principle [55, 56], the volumes of the CGM, area 24 and layer V in area 24 were estimated. The profile areas of the CGM were determined using point counting [55], whereas the profile areas of area 24 as well as layer V in area 24 were obtained by reading off the calculated area in the software’s contour measurements. The actual section thickness after the histologic procedure was determined as described in Heinsen et al. [57] and varied between 455 and 578 µm.

Total neuron numbers were determined using the optical fractionator method [58]. In that procedure, a series of unbiased virtual counting spaces (UVCSs) covers the ROI. The ROI-specific size of the UVCSs and the distance in X and Y directions between the UVCSs were determined in pilot studies, such that a per-hemisphere count of approximately 500 objects of interest (neurons and VENs) was achieved. This procedure resulted in low coefficients of error (CE < 0.1) [59, 60].

**Fig. 3:**
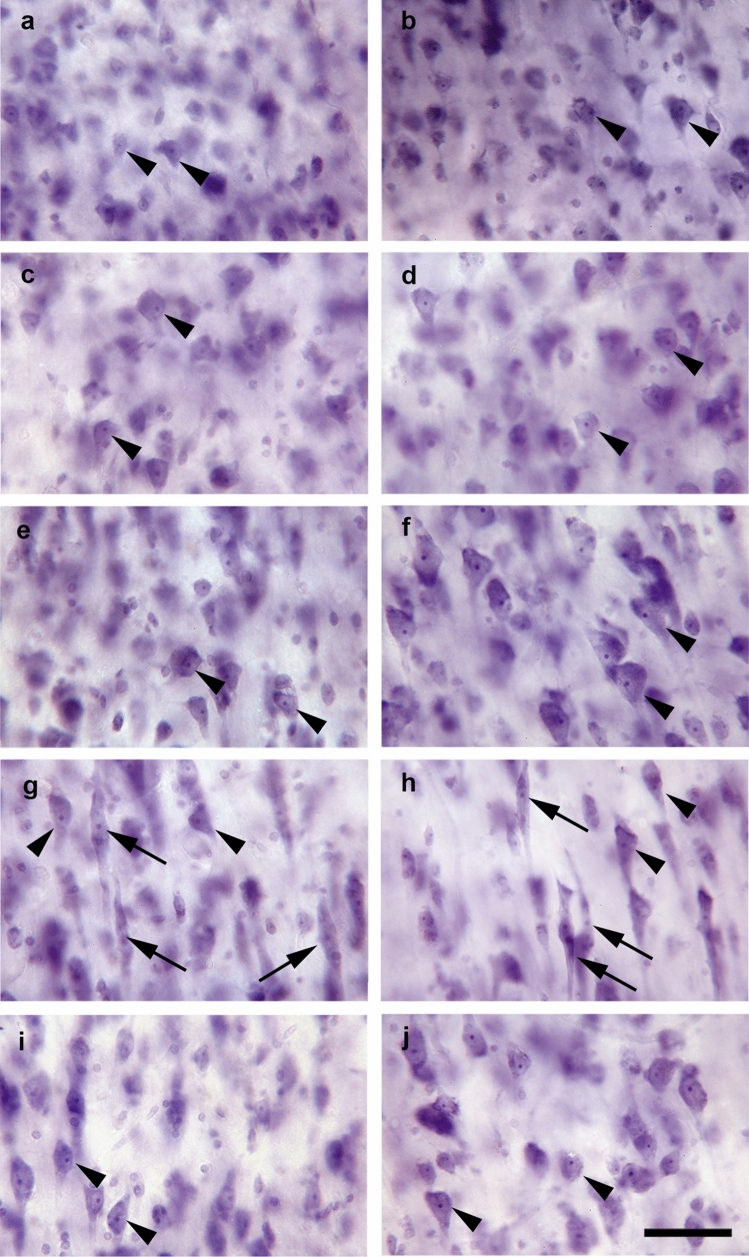
Representative photomicrographs of different layers in area 24. The panels show layers II (**a**, **b**), III (**c**, **d**), Va (**e**, **f**), Vb (**g**, **h**) and VI (**i**, **j**) in a hemisphere from a control subject (**a**, **c**, **e**, **g**, **i**) and a patient with schizophrenia (**b**, **d**, **f**, **h**, **j**). Arrowheads point to neurons (predominantly pyramidal cells) and arrows to VENs. Scale bar = 50 µm in **a**–**j**

Neurons and VENs were counted at high magnification (20 × and 40 × objectives); further details of the stereologic counting procedure are provided in Supplementary Table 1. Identification of neurons was based on their typical shape: a large soma with several dendrites emerging from the soma and a prominent dark nucleolus (Fig. 3). VENs were identified by an elongated soma, basal and apical dendrite, distinct ovoid nucleus and prominent dark nucleolus (Fig. 3g, h). Neurons and VENs were counted in case their nucleolus came into focus within an UVCS and did not hit the exclusion line (red in Supplementary Fig. 1) or hit the inclusion line (green in Supplementary Fig. 1) of the unbiased counting frame [46]. Neuron densities were calculated by dividing the estimated total neuron number of a given ROI by the estimated total volume of this ROI.

### Statistical analysis

An adjusted between-group Cohen’s d effect size (*d*_adj_) and adjusted percentage group difference were calculated for each of the investigated outcome variables. The adjustment for extraneous variables was carried out by constructing a separate linear model (analysis of covariance, ANCOVA) with each outcome as the independent variable, in turn. The model included the fixed factors diagnosis (control vs. schizophrenia) and hemisphere, and the covariates age, postmortem interval and fixation time (five degrees of freedom). Treatment coding was used for the fixed factor diagnosis, with the control group as reference while sum coding was used for hemisphere. The covariates were mean-centered prior to fitting for better interpretability of the intercept.

*P* values were obtained from the *t* statistic of ANCOVA for each fixed factor and covariate. The statistical significance level alpha was set to 0.05. *d*_adj_ values were computed from the coefficients of the diagnosis fixed factor in each model. [61] Consistent with Cooper et al. [61], effect sizes in the ranges 0.2–0.5, 0.5–0.8, and above 0.8 were interpreted as small, medium and large, respectively. Of note, percentage group differences were also adjusted by dividing the coefficient of the fixed factor diagnosis by the model intercept (by design, the model intercept represented the outcome’s mean in the control group after adjustment by hemisphere and covariates). Lastly, all subjects’ outcome values (volumes, neuron numbers and neuron densities) were adjusted by the covariates age, postmortem interval and fixation time. This was achieved by subtracting the estimated linear effect of the covariates from the raw value. These adjusted outcome values were visualized for each diagnosis and hemisphere group via box plots. The statistical analysis was performed using the Python libraries pandas [62], statsmodels [63] and SciPy [64]. The graphical visualization was performed with GraphPad Prism (version 9 for Windows, GraphPad Software, San Diego, CA, USA).

## Results

**Fig. 4:**
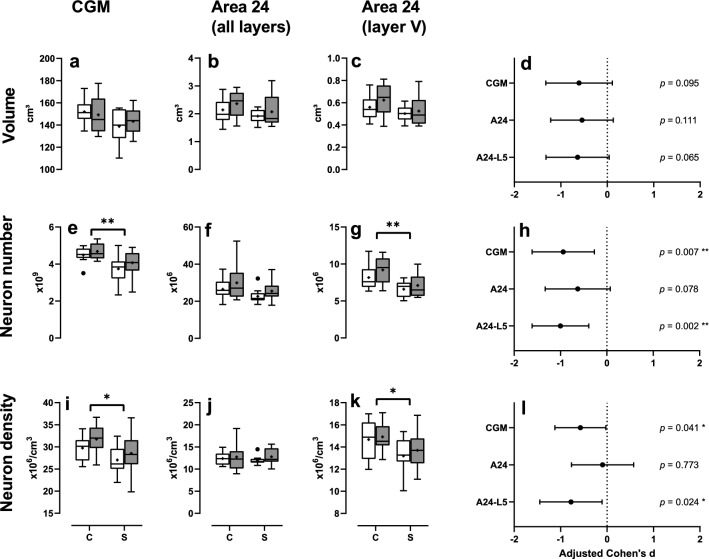
Results of the design-based stereologic analyses, Part 1: The panels show Tukey box plots of total volume (**a**–**c**), total neuron number (**e**–**g**) and neuron density (**i**–**k**) in whole CGM (**a**, **e**, **i**), area 24 (all layers) (**b**, **f**, **j**) and layer V of area 24 (**c**, **g**, **k**) after adjustment by covariates in the left cortical hemisphere (open boxes) and right cortical hemisphere (grey boxes) of patients with schizophrenia (S) and matched controls (C), as well as 95% confidence intervals of adjusted Cohen’s *d* effect sizes (*d*_adj_) for each outcome (**d**, **h**, **l**). Statistically significant results are indicated (*P < 0.05; **P < 0.01). *CGM* cortical gray matter

No differences in mean volumes of the investigated ROIs were observed between patients with schizophrenia and controls. The mean CGM volume was 6.3% smaller in patients with schizophrenia than in controls (after adjustment by hemisphere and the covariates age, postmortem interval and fixation time) and had an adjusted Cohen’s d of *d*_adj_ =  − 0.61 (*P* = 0.095; Fig. 4a). Comparable results were obtained for the volumes of area 24 (on average − 11.6%, *d*_adj_ =  − 0.54, *P* = 0.111; Fig. 4b) and layer V of area 24 (on average − 13.2%, *d*_adj_ =  − 0.64, *P* = 0.065; Fig. 4c). Corresponding adjusted Cohen’s d effect sizes are given in Fig. 4d. Results of statistical analysis (*P* values of ANCOVA covariates and fixed factors) are summarized in Table 2.

**Table 2:** Results of statistical analysis: *P* values of ANCOVA covariates and fixed factors

Region of interest	Variable	Age	PMI	Fix	Diagnosis	Hemisphere
CGM	Volume	0.072	0.090	0.480	0.095	0.802
CGM	Neuron number	**0.035**	** < 0.001**	0.064	**0.007**	0.179
CGM	Neuron density	** < 0.001**	** < 0.001**	0.070	**0.041**	0.127
Area 24	Volume	0.435	0.734	0.200	0.111	0.173
Area 24	Neuron number	0.696	0.409	0.265	0.078	0.101
Area 24	Neuron density	0.097	0.365	**0.007**	0.773	0.295
Area 24 Layer V	Volume	0.693	0.502	0.377	0.065	0.221
Area 24 Layer V	Neuron number	0.442	0.057	0.603	**0.002**	0.113
Area 24 Layer V	Neuron density	**0.041**	**0.025**	0.398	**0.024**	0.434
Area 24 Layer V	VEN number	0.799	0.917	0.084	**0.027**	0.842
Area 24 Layer V	VEN density	0.844	0.504	0.171	0.128	0.836

*P* values < 0.05 are given boldface

*PMI* postmortem interval, *Fix* fixation time, *CGM* cortical gray matter, *VEN* von Economo neuron

The mean total neuron number in CGM was lower in patients with schizophrenia than in controls (− 14.9%, *d*_adj_ =  − 0.94, *P* = 0.007, Fig. 4e). No difference was observed in the mean total neuron number of area 24 between patients with schizophrenia and controls (− 14.5%, *d*_adj_ =  − 0.63, *P* = 0.08; Fig. 4f). However, the mean total neuron number in layer V of area 24 was lower in patients with schizophrenia than in controls (− 21.1%, *d*_adj_ =  − 1.00, *P* = 0.002, Fig. 4g). Corresponding adjusted Cohen’s d effect sizes are given in Fig. 4h.

Compared to controls, patients with schizophrenia showed a lower mean neuron density in CGM (− 9.63%, *d*_adj_ =  − 0.57, *P* = 0.041; Fig. 4i) but not in the whole area 24 (− 1.61%, *d*_adj_ =  − 0.10, *P* = 0.773; Fig. 4j). Nevertheless, the mean neuron density in layer V of area 24 was lower in patients with schizophrenia than in controls (− 9.08%, *d*_adj_ =  − 0.78, *P* = 0.024; Fig. 4k). Corresponding adjusted Cohen’s d effect sizes are given in Fig. 4l.

**Fig. 5:**
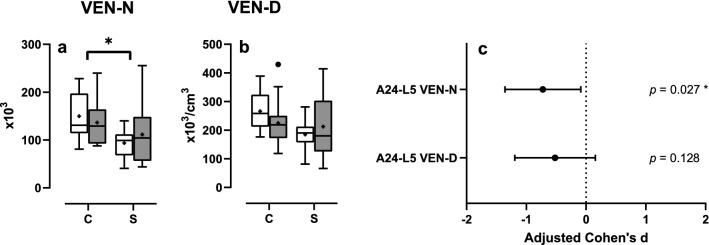
Results of the design-based stereologic analyses, Part 2: The panels show Tukey box plots of the estimated total number of VENs (**a**) and the estimated VEN density (**b**) in layer V of area 24 after adjustment by covariates in the left cortical hemisphere (open boxes) and right cortical hemisphere (grey boxes) of patients with schizophrenia (S) and matched controls (C), as well as 95% confidence intervals of adjusted Cohen’s *d* effect size for each outcome (**c**). Statistically significant results are indicated (*, *P* < 0.05; **, *P* < 0.01). *VEN* von Economo neuron

The mean total number of VENs in layer V of area 24 was, as well as the mean total neuron number, lower in patients with schizophrenia than in controls (− 28.3%, *d*_adj_ =  − 0.72, *P* = 0.027, Fig. 5a). There was no difference between patients with schizophrenia and controls in the mean density of VENs in layer V of area 24 (− 19.06%, *d*_adj_ =  − 0.52, *P* = 0.128; Fig. 5b). Corresponding adjusted Cohen’s d effect sizes are given in Fig. 5c.

Among the covariates, age had an effect on the total neuron number in CGM (ANCOVA coefficient: + 0.028 × 10^9^ per year of age, *P* = 0.035), neuron density in CGM (+ 0.313 × 10^6^/cm^3^ per year of age, *P* < 0.001) and neuron density in layer V of area 24 (+ 0.067 × 10^6^/cm^3^ per year of age, *P* = 0.041). Furthermore, the postmortem interval had an effect on neuron number in CGM (+ 0.024 × 10^9^ per hour PMI, *P* < 0.001), neuron density in CGM (+ 0.221 × 10^6^/cm^3^ per hour PMI, *P* < 0.001) and neuron density in layer V of area 24 (+ 0.034 × 10^6^/cm^3^ per hour PMI, *P* = 0.025). A singular effect of the fixation time on the neuron density in area 24 (− 0.001 × 10^6^/cm^3^ per day in fixative, *P* = 0.007) was observed, as well. Because these covariates were included in the ANCOVA model and thus statistically controlled for, the observed differences in mean CGM neuron number and mean area 24 layer V neuron density in patients with schizophrenia were not attributable to differences in patients’ age or postmortem interval. In this regard, no obvious trends were also evident in graphical diagrams of the respective outcome variables against these covariates (Supplementary Figs. 2, 3). Finally, no other effects of covariates or hemisphere on the different outcomes were present, meaning there were also no hemispheric asymmetries in any outcome.

There was no difference in individual ratios of mean total neuron numbers in layer V of area 24 and the lateral amygdaloid nucleus between patients with schizophrenia and controls (*d*_adj_ =  − 0.19, *P* = 0.599; Supplementary Fig. 4; the total neuron numbers in the lateral amygdaloid nucleus in the same brains investigated in the present study were previously determined by Kreczmanski et al. [46]). Moreover, the correlation between individual total neuron numbers in layer V of area 24 and the lateral amygdaloid nucleus was positive and of moderate strength in all subjects (*r* = 0.407, *P* = 0.005, Supplementary Fig. 5A) and positive and of weak strength in the control (*r* = 0.226, *P* = 0.313, Supplementary Fig. 5B) and patient (*r* = 0.264, *P* = 0.212, Supplementary Fig. 5C) groups, respectively.

The full statistical analysis was repeated without the data of the 22-year-old patient with schizophrenia (S01), for whom there was no age-matched control case. This resulted in no change in the magnitudes of adjusted Cohen’s d effect sizes and in the significance of p values (Supplementary Figs. 6, 7).

## Discussion

### Lower mean CGM neuron number and density in patients with schizophrenia

The estimated mean CGM volume, mean total CGM neuron number and mean CGM neuron density of the controls reported in this study are in line with previous reports [47, 65]. To our knowledge, only one study [66] compared the mean total neuron number in the whole cerebral cortex between patients with schizophrenia and controls. In that study [66], no statistically significant difference between the groups was found. However, while patients with schizophrenia and controls were matched for sex and age, other important extraneous variables were not statistically accounted for. Our data indicate that the postmortem interval has a statistically significant effect on the estimated CGM total neuron number and neuron density.

Meta-analyses of structural MRI studies of smaller mean cortical volume in patients with schizophrenia compared to controls have reported Cohen’s d effect sizes of − 0.43 and − 0.28/ − 0.27 (for left/right hemisphere) [4, 8]. A recent large-scale meta-analysis [10] observed effect sizes of − 0.530/ − 0.516 (left/right hemisphere) for mean cortical thickness and − 0.251/ − 0.254 for mean cortical surface area, implying a lower mean cortical volume, as well. Other studies [4, 7] focused on specific brain lobes and found widespread indications of smaller mean cortical volume, with an emphasis on the temporal and frontal lobes. Such findings extend also to the ACC [6, 10, 12, 13, 67] with meta-analytic effect size estimates of − 0.34 and − 0.26 between patients with schizophrenia and controls [8, 9].

Notably, the effect sizes from these meta-analyses are of comparable magnitude to the effect sizes *d*_adj_ of the present study for patients with schizophrenia vs. control regional volume differences. However, our results did not reach statistical significance.

Our observation that the mean CGM neuron density is significantly lower in patients with schizophrenia than in controls contrasts with evidence from meta-analyses demonstrating increased [68] or unchanged [69] mean cortical neuron density in patients with schizophrenia. However, these meta-analyses must be interpreted with some caution, because they aggregated studies that analyzed individual cortical regions and not the whole cerebral cortex. Based on an analysis of whole-cortex CGM, our finding of a significantly lower mean CGM neuron density suggests that the smaller mean CGM neuron number in patients with schizophrenia is not merely a direct consequence of a decreased CGM volume but that it reflects a superimposed disease process disproportionately affecting cortical neurons. Because we assessed only the total neuron number in the whole CGM, it remains unclear whether this represents a diffuse process over the whole cerebral cortex or localized to a few specifically affected brain regions (at least area 24 was not affected).

### No statistically significant differences in area 24 between patients with schizophrenia and controls

We did not observe statistically significant differences in mean area 24 total neuron number and mean area 24 volume between patients with schizophrenia and controls and, consequently, no statistically significant differences in mean neuron density. These findings add to the growing evidence of unchanged area 24 neuron density in patients with schizophrenia [23, 28, 30–32]. Two studies [25, 26] found no change in mean pyramidal neuron density as well, and statistically significant but opposed differences in interneuron density. Also consistent with previous work is our finding that the mean area 24 total neuron number did not differ between patients with schizophrenia and controls [22, 32]. Altogether, these results suggest that area 24 as a whole is not affected by the pathological process that results in a decreased mean CGM total neuron number in schizophrenia.

### Lower mean area 24 layer V total neuron number and neuron density in patients with schizophrenia

Layer V of area 24 stood out from the overall unaffected area 24, with statistically significant differences in mean total neuron number and mean neuron density between patients with schizophrenia and controls. This selective reduction of the mean total neuron number in layer V of area 24 was not visible in the whole area 24, as the mean total neuron number of layer V constituted only 30% of the mean total neuron number of area 24.

Our findings replicate an earlier finding [28] of a lower neuron density specifically in layer V of area 24 in patients with schizophrenia, while neuron densities in all other layers of area 24 were not affected [28]. Another study [26] reported a significantly lower mean interneuron density in area 24 layer V in patients with schizophrenia, but no change in the mean density of pyramidal neurons. Other studies failed to find such differences [23, 25, 30] or described a higher layer V neuron density in patients with schizophrenia [29]. Our findings suggest that layer V of area 24 plays a specific role in the pathophysiology of schizophrenia.

Area 24 is involved in the regulation of emotional and cognitive functions [70–72] and is extensively connected to many cortical, subcortical and spinal regions [73–77]. A large part of the efferent portion of these connections originate in layer V of area 24 [78, 79]. It is therefore likely that a smaller mean total neuron number in this layer impairs area 24 functional connectivity output in schizophrenia, as suggested by a few functional neuroimaging studies [17–19]. There is in fact solid evidence of abnormalities of the cingulum bundle in schizophrenia [80–82], a large fiber tract that carries the major portion of connections to and from area 24 [83]. One important target of efferent area 24 projections is the amygdala, and specifically its lateral and basolateral nuclei [77, 78, 84]. An earlier investigation [46] of the same brains that were analyzed in this study found a significantly lower mean total neuron number in the lateral nucleus of the amygdala in patients with schizophrenia compared to controls. Indeed, when we included the original data from that study [46] in our analysis, we found that the total numbers of neurons in layer V of area 24 and in the lateral amygdaloid nucleus were equally lowered in patients with schizophrenia compared with controls, and that there was a positive (albeit weak) correlation between the total number of neurons in these two regions in both the control and patient groups. This suggests that projections from area 24 to the amygdala are impaired in schizophrenia, which is in line with results from neuroimaging findings indicating abnormal connectivity between the amygdala and the ACC [85]. Furthermore, connectivity studies demonstrated a trend toward reductions in connectivity between brain areas in schizophrenia [18], which was also commonly seen in the ACC [19]. Other postmortem studies demonstrated potential dysfunction in the GABA-ergic and glutamatergic systems, leading to Benes’ hypothesis of a disturbed prefrontal cortical—anterior cingulate—lateral amygdaloid nucleus—hippocampal circuit [86]. Our results further support this hypothesis that may explain key factors of the pathogenesis and clinical features of cardinal symptoms of schizophrenia.

### Lower mean von Economo neuron number in layer V of area 24 in patients with schizophrenia

The significantly lower mean total neuron number in layer V of area 24 in patients with schizophrenia was accompanied by a lower mean total number of VENs. The mean VEN density, however, did not differ between patients with schizophrenia and controls.

Prior research on the neuropathology of VENs in schizophrenia is sparse. One study [42] discovered more lysosomal aggregations in VENs in patients with schizophrenia compared to controls, suggesting aberrations at the single cell level. Consistent with our results, Brüne et al. [21] found no difference between patients with schizophrenia and healthy controls when comparing the mean density of VENs in layer V of area 24, but reported a significantly lower mean VEN density specifically in the right ACC of four patients with early-onset schizophrenia. Because a design-based stereologic approach was not applied by Brüne et al. [21], a possible difference in the mean total VEN number between the groups was not assessed. Rigorous, design-based stereology on tissue sections sampled systematically and randomly from the entire extent of the ROI produces the most valid and reliable estimates of ROI volume and total neuron numbers, and should be used as a standard in quantitative neurohistology [59, 87]. There is evidence for selective reduction in the mean ACC VEN number in neuropsychiatric disorders characterized by severe deficits in social cognition such as frontotemporal dementia [41], agenesis of the corpus callosum [40] and autism spectrum disorder [33, 88], while no such selective reduction occurs in other conditions in which social cognitive defects do not play a central role, such as Alzheimer’s disease [41]. Such findings have led to the concept that the VENs in the ACC may play a critical role in social cognitive functioning [33], further supported by substantial evidence for a role of the ACC in social cognition [89, 90]. Deficits in social cognition are a well-known symptom of schizophrenia as well [91], and as such alterations in the number of VENs may play a substantial role in the pathophysiology of schizophrenia [33]. While definite conclusions certainly cannot be drawn due to a scarcity of data, the results of this study lend further support to this hypothesis.

## Limitations

While the effects of a number of important extraneous variables were controlled for, our results were not adjusted for antipsychotic medication exposure because such data were not available. The same applied to other clinical characteristics such as IQ and other medication.

As most neuropathologic studies, this study is constrained by a small sample size, leading to large confidence intervals on effect sizes. Furthermore, determining subgroups in patients with schizophrenia was not possible as this would have resulted in too small sample sizes (e.g., three of twelve patients with schizophrenia committed suicide).

Another limitation is the fact that our study only included males and did not address possible sex differences. In fact, sex differences were reported for several variables in schizophrenia, including incidence, age of onset, symptoms and brain structure. Overall, males show a higher incidence than females (male/female incidence 1.4:1) [92]. Furthermore, males tend to have an earlier onset and worse negative symptoms compared to females [93]. There are reports about sex-specific effects on brain development and, although inconsistent, about sex-specific structural brain differences in schizophrenia, with males showing overall greater structural abnormalities than females [66, 93, 94]. Different sex differentiation of the brain may be linked to sex-specific abnormalities in schizophrenia. Women may be more protected against the development of schizophrenia than men because of the hypothesized protective effect of estrogen on the brain of women [93, 94]. Neuropathologic studies with larger sample sizes, considering both sexes, will fill the gap of knowledge regarding sex differences at the cellular level in schizophrenia.

We did not perform separate subanalyses of regions 24a, 24b and 24c. The latter would require ideal perpendicular sections through the cingulate gyrus since cytoarchitectonic parcellation is based on subjective estimation of neuronal cell size, density, shape and relative laminar thickness of cortical layers, and tangential sections would inevitably distort parcellation. Ideal perpendicular sections to the pial surface are rarely encountered in serial coronal sections of whole human brain hemispheres and complicate a thorough subdivision of area 24.

Finally, the fact that all brain hemispheres in our sample of postmortem brains of patients with schizophrenia and controls were cut into complete series of coronal sections precluded testing of the hypothesis of impaired projections from area 24 to the amygdala. The latter would require tracing experiments that are not possible anymore after cutting entire hemispheres into tissue sections [86].

## Conclusion and future directions

Our data indicate a global disease process affecting cortical neurons in schizophrenia. Area 24 taken as a whole is apparently not affected by this disease process. However, within area 24, layer V showed specific deficits in total neuron and VEN numbers, which likely contribute to an impaired ACC connectivity in schizophrenia. Future postmortem studies could focus on the hypothesis of an impaired ACC connectivity in context with other regions such as amygdala and provide further insight.

There is evidence of [49] multiple biological subtypes of schizophrenia with differing neuroanatomic presentations [95–97], which could partly explain the discrepancy of results from neuropathologic studies. Accordingly, analyses of group-level differences in brain structure reporting averages might not capture the complexity of the disorder and should be supplemented with assessment of individual patterns of brain pathology [98]. This can be realized by repeated analyses of the same postmortem whole-brain samples at many anatomic sites, resulting in a more comprehensive microscopic evaluation of pathology. Neuropathology can, in this way, complement psychiatric neuroimaging research with critical information at cell-level resolution.

## Supplementary Information

Below is the link to the electronic supplementary material.

Supplementary file1 (DOCX 2004 KB)

## Data Availability

The data that support the findings of this study are openly available in G-Node at https://doi.org/10.12751/g-node.rovhrd [99].
