# Supplementary material for: Reduced cortical neuron number and neuron density in schizophrenia with focus on area 24: a post-mortem case–control study
Source: Eur Arch Psychiatry Clin Neurosci. 2022 Nov 9;273(6):1209–23. doi: 10.1007/s00406-022-01513-6 (PMC10449727; doi:10.1007/s00406-022-01513-6)
Supplement: Supplementary file 1 — Supplementary file1 (DOCX 2004 KB) [file 406_2022_1513_MOESM1_ESM.docx]

**Supplementary Information**

**Reduced cortical neuron number and neuron density in schizophrenia with focus on area 24: a post-mortem case-control study**

*European Archives of Psychiatry and Clinical Neuroscience*

Richard Gaus, Melanie Popal, Helmut Heinsen, Andrea Schmitt, Peter Falkai, Patrick R. Hof, Christoph Schmitz and Alisa Vollhardt

**Correspondence to:** Alisa Vollhardt

Research associate at Department of Neuroanatomy, Institute of Anatomy, Faculty of Medicine, LMU Munich, Pettenkoferstr. 11, 80336 Munich, Germany

alisa.vollhardt@med.uni-muenchen.de

**Content**

**This file includes:**

- Supplementary Methods
- Supplementary Table 1
- Supplemantary Table 2
- Supplementary Figure 1
- Supplementary Figure 2
- Supplementary Figure 3
- Supplementary Figure 4
- Supplementary Figure 5
- Supplementary Figure 6
- Supplementary Figure 7
- Supplementary References

# Supplementary Methods

## **Photography**

Photographs of the whole hemisphere, area 24 and individual layers in area 24 were taken with a Canon EOS 5D Mark III (Canon, Tokyo, Japan) camera. For the overview pictures of the hemispheres, a Canon Macro Lens EF 100 mm objective was utilized, while a Canon Macro Photolens MP-E 65 mm objective was used for the overview pictures of area 24. All overview pictures were taken using a universal camera mount (Polaroid MP-4 Land Camera, Polaroid Corporation, Minnetonka, Minnesota, USA) and a LED light table (Kaiser Slimlite, Kaiser Fototechnik, Buchen, Germany). For the high-power photomicrographs of the different layers in area 24 (Fig. 3 in the main paper), the Canon camera was set up with an Olympus BH2 microscope (Olympus, Tokyo, Japan), using a Zeiss Planapo objective 40 (NA=0.95). Post-production of the photographs (merging, adjustment of contrast and brightness) was executed with Adobe Photoshop CS 6 (Adobe, San Jose, CA, USA). Care was taken that the original appearance of the material remained unchanged.

|  | **CGM** | **Area 24 All layers** | **Area 24 Layer V** | **Area 24 Layer V: VEN** |
| --- | --- | --- | --- | --- |
| Obj | 40× | 20× | 20× | 20× |
| B [µm^2^] | 1156 | 1764 | 1764 | 40,000 |
| H [µm] | 30 | 50 | 50 | 50 |
| D [µm] | 5000 | 1200 | 600 | 400 |
| ∑ N_sections_ | 10 | 13 | 13 | 13 |
| ∑ UVCS | 588 | 615 | 679 | 2023 |
| ∑ Q^-^ | 514 | 614 | 769 | 614 |
| CE_pred_ [n] | 0.044 | 0.041 | 0.038 | 0.041 |
| ∑ P (Cavalieri) | 512 | - | - | - |

**Supplementary Table 1: Details of the stereologic counting procedure**

**CGM**, cortical gray matter; **VEN**, von Economo neurons; **Obj**, objective used; **B** and **H**, base and height of the unbiased virtual counting spaces; **D**, distance between the unbiased virtual counting spaces in orthogonal directions *x* and *y*; **∑ N_sections_**, average number of sections analyzed per hemisphere; **∑UVCS**, average number of unbiased virtual counting spaces used; **∑Q^-^**, average number of counted neurons; **CE_pred_ [n]**, average predicted coefficient of error of the estimated total neuron numbers using the prediction method described by Schmitz ^1^ and Schmitz and Hof ^2^; **∑P**, average number of counted points within the ROI (for estimation of CGM volume).

| Stereology workstation No.1 | - Zeiss Axioskop microscope (Carl Zeiss Microscopy, Jena, Germany) - Zeiss Plan-Neofluar objectives   - 1.25× (numerical aperture [NA] = 0.035)   - 2.5× (NA = 0.075)   - 10× (NA = 0.30)   - 20× (NA = 0.50)   - 40× (NA = 0.75)   - 100× (NA = 1.30 oil) - incremental length gauge (Type MT 1271; Heidenhain, Traunreut, Germany) - stage controller (Type MAC 6000; Ludl Electronics Products, Hawthorne, NY, USA) - 12 bit Color Camera (1,600 x 1,200 pixels; Model 01-MBF-2000R-F-CLR12; MBF Bioscience, Williston, VT, USA) - stereology software (Stereo Investigator Version 11.01.2 64 bit; MBF Bioscience) |
| --- | --- |
| Stereology workstation No.2 | - Zeiss Axio Imager M2 microscope (Carl Zeiss Microscopy) - Zeiss EC Plan-Neofluar objectives   - 1.25× (NA = 0.03), 2.5× (NA = 0.085)   - 100× (NA= 1.30, oil) - Zeiss Plan-Apochromat objectives   - 5× (NA = 0.16), 10× (NA = 0.45)   - 20× (NA = 0.8)   - 40× (NA = 0.95) - stage controller (Type MAC 6000; Ludl Electronics Products) - Luminar HR Camera (2,752 x 2,192 pixels; Model: Lt665RC-MBF, MBF Bioscience) - stereology software (Stereo Investigator Version 2018.2.2 64 bit; MBF Bioscience) |

**Supplementary Table 2: Details on the stereology workstations**

Equipment of the stereology workstations used for analyses. Two investigators performed stereologic analyses using the stereology workstation No. 1. The third researcher performed stereologic analyses on stereology workstation No.2.


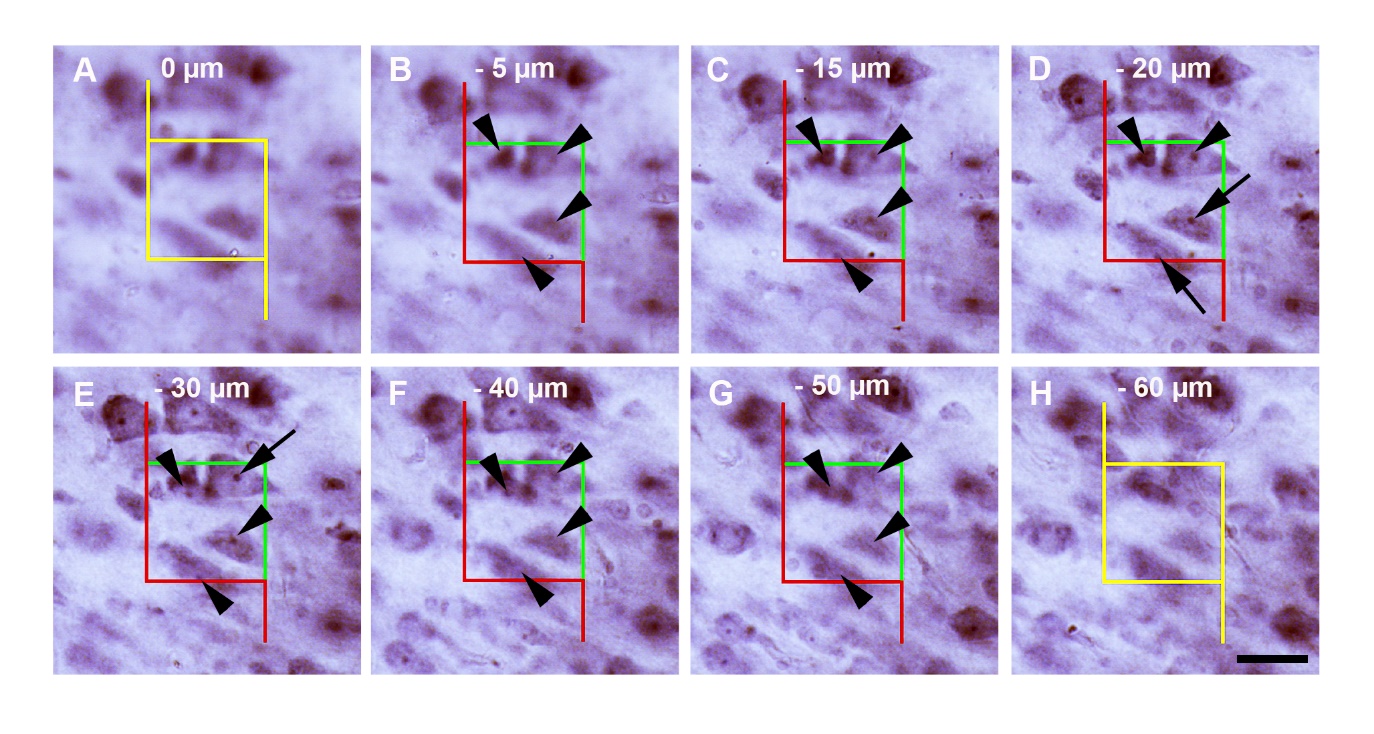


**Supplementary Fig.1: Counting procedure with unbiased virtual counting frames in area 24** The panels show the counting procedure for neurons in area 24 from a control subject using unbiased counting frames (42 µm for width and length) at each focal plane, representing the base area of an unbiased virtual counting space with a height of 50 µm (**B**-**G**). Neurons were counted whose nucleolus came into focus within the unbiased virtual counting spaces and did not hit the exclusion line (red) or hit the inclusion line (green) of the unbiased virtual counting frames. Panels **D** and **E** show neurons with their nucleolus in focus (arrows), fulfilling all counting criteria. In contrast, the arrowheads indicate neurons whose nucleolus are not in focus and therefore do not fulfill all counting criteria (**B-G**). In total, the investigator should count three neurons in this example of the design-based stereologic counting procedure (two in panel **D**, one in panel **E**). The yellow frames indicate that the corresponding focal plane was either above (**A**) or below (**H**) the unbiased virtual counting space. The numbers in **B** to **H** indicate the distance from the top of the section. Scale bar = 25 µm in **A**-**H**.


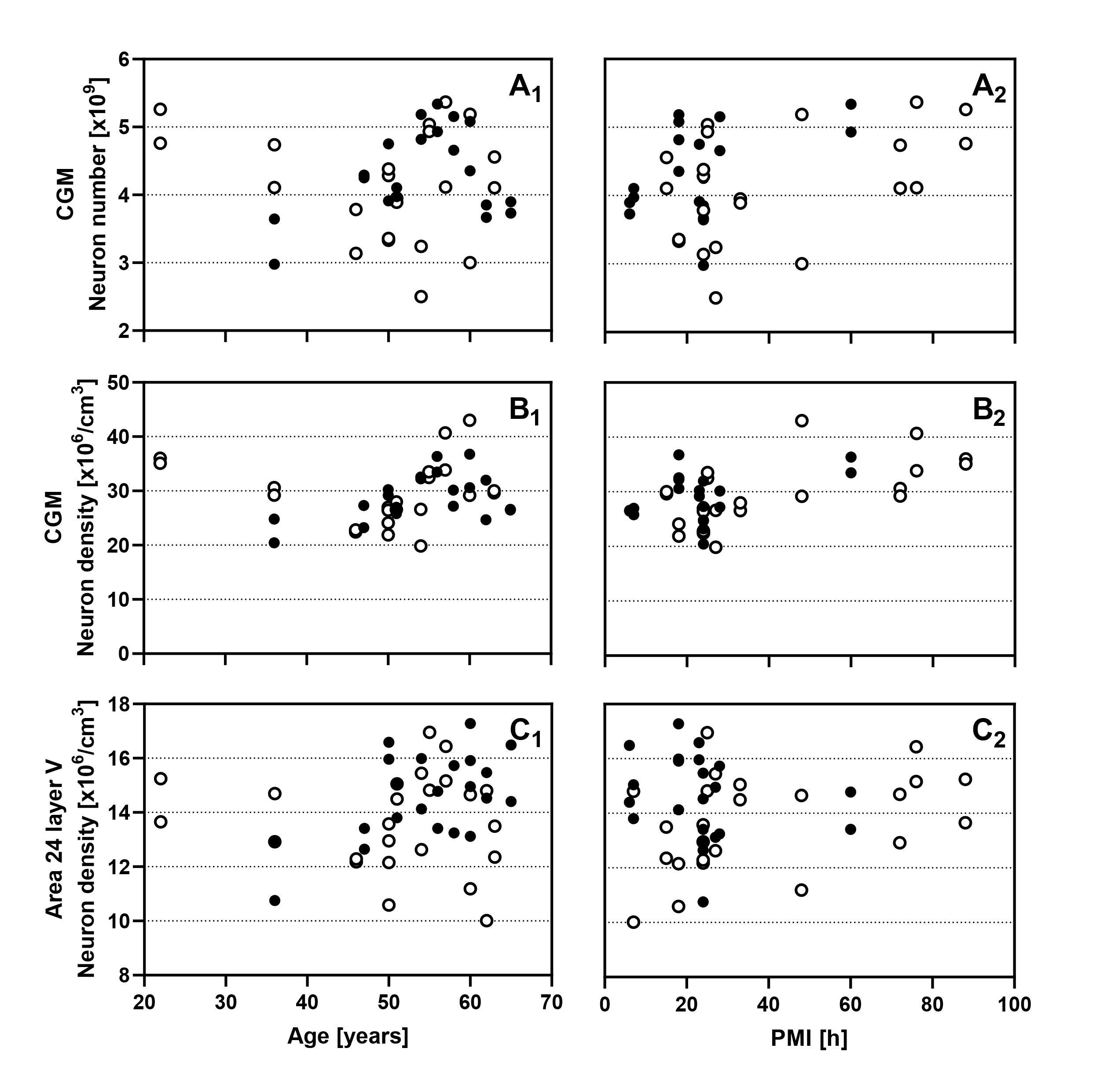


**Supplementary Fig. 2: Outcome variables as functions of covariates, Part 1** The panels show the individual CGM total neuron number (**A1**, **A2**), CGM neuron density (**B1**, **B2**) and area 24 layer V neuron density (**C1**, **C2**) as a function of age (**A1**, **B1**, **C1**) as well as a function of the post mortem interval (PMI) (**A2**, **B2**, **C2**). Values from controls are given as closed dots and values of patients with schizophrenia as open dots.

In the analysis of covariance, age had an effect on the total neuron number (P = 0.035) and neuron density (P < 0.001) in CGM, and neuron density in layer V of area 24 (P = 0.041). In addition, the postmortem interval had an effect on neuron number (P < 0.001) and neuron density (P < 0.001) in CGM, and neuron density in layer V of area 24 (P = 0.025).


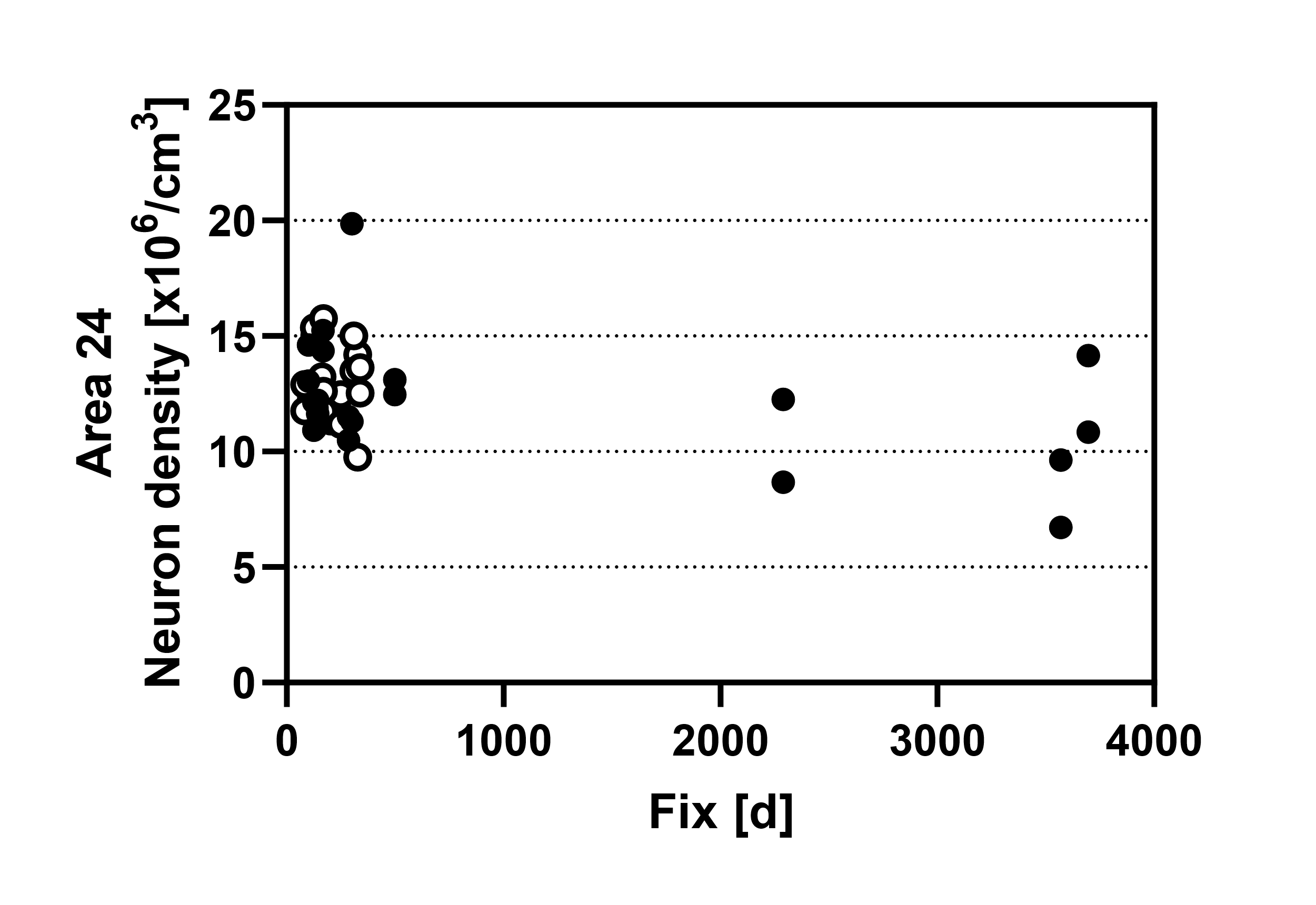


**Supplementary Fig. 3: Outcome variables as functions of covariates, Part 2** The figure shows the individual area 24 neuron density as a function of the fixation time. Values from controls are given as closed dots and values of patients with schizophrenia as open dots.

In the analysis of covariance, the fixation time had an effect on the neuron density in area 24 (P = 0.007).


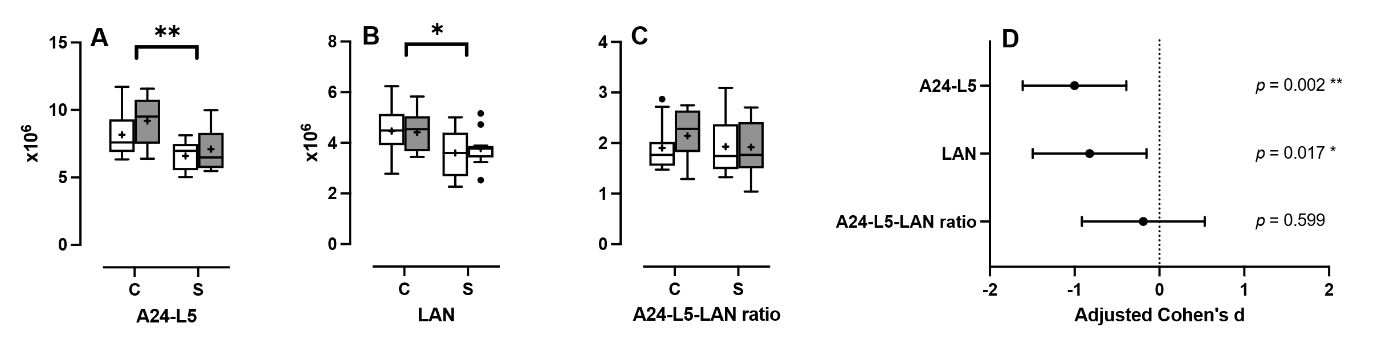


**Supplementary Fig. 4: Comparison of neuron numbers in layer V of area 24 and lateral amygdaloid nucleus, Part 1** The panels show Tukey box plots of total neuron number in layer V of area 24 (**A**) and the lateral amygdaloid nucleus (**B**), and the ratio of total neuron numbers in these two regions (**C**) after adjustment by covariates (age, post-mortem interval, fixation time) in the left cortical hemisphere (open boxes) and right cortical hemisphere (grey boxes) of patients with schizophrenia (**S**) and matched controls (**C**), as well as 95% confidence intervals of adjusted Cohen’s d effect sizes for each outcome (**D**). Raw data on LAN neuron numbers was taken from Kreczmanski et al. ^3^ Statistically significant results are indicated (*, *P* < 0.05; **, *P* < 0.01). **A24-L5**, layer V of area 24; **LAN**, lateral amygdaloid nucleus; **A24-L5-LAN ratio**, Ratio between total neuron number in A24-L5 and LAN.


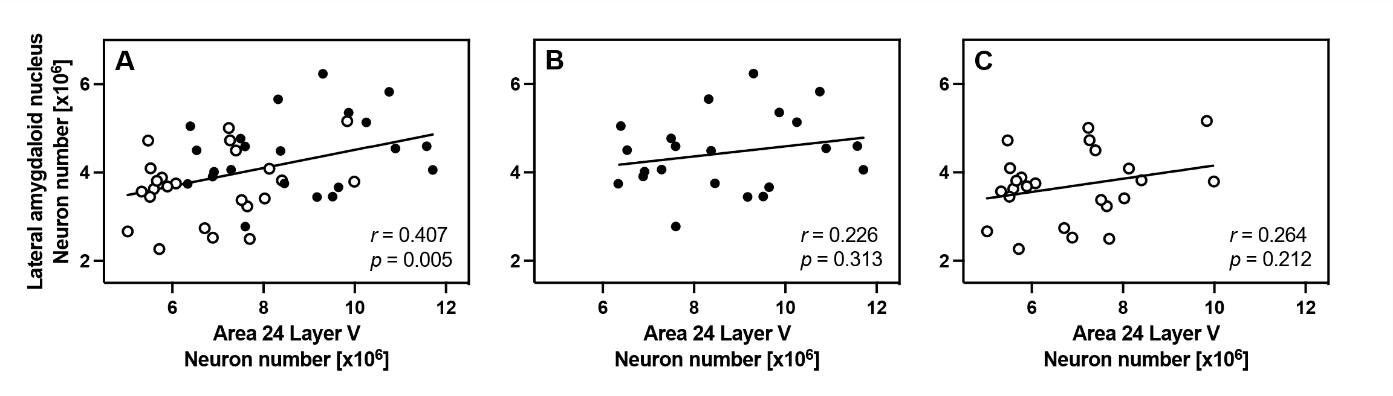


**Supplementary Fig. 5: Comparison of neuron numbers in layer V of area 24 and lateral amygdaloid nucleus, Part 2** The panels show mean total neuron number in the lateral amygdaloid nucleus as a function of mean total neuron number in layer V of area 24 in all subjects (**A**), controls only (**B**), and patients with schizophrenia only (**C**). Values from controls are given as closed dots and values of patients with schizophrenia as open dots. Neuron numbers are adjusted by covariates. Least squares regression lines are given in black. Raw data on LAN neuron numbers was taken from Kreczmanski et al. ^3^ ***r***, Pearson’s correlation coefficient; ***p***, p-value of correlation analysis.

**
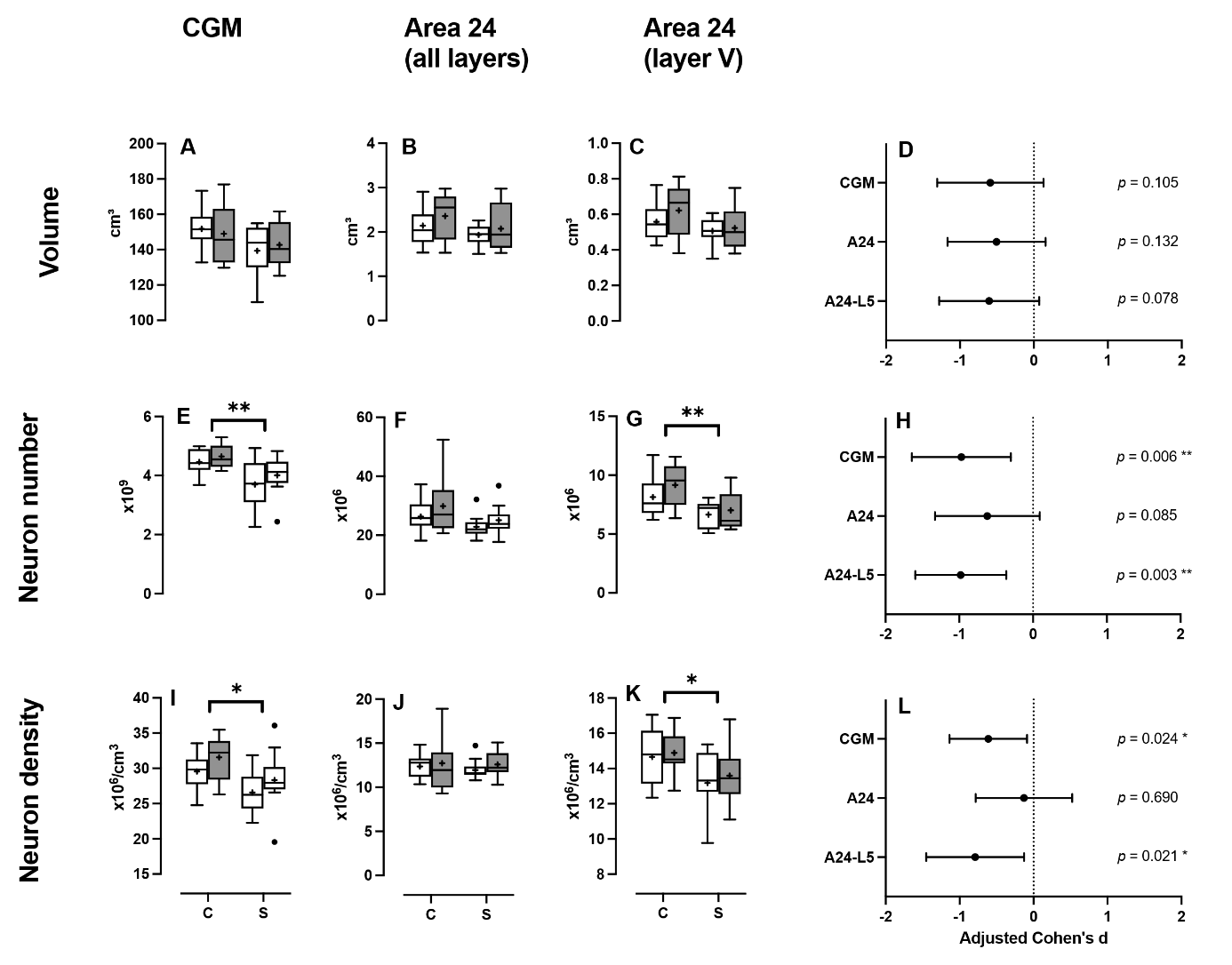
**

**Supplementary Fig. 6: Results of the design-based stereologic analyses after omitting patient S01, Part 1** The panels show Tukey box plots of total volume (**A**-**C**), total neuron number (**E**-**G**) and neuron density (**I**-**K**) in whole CGM (**A**, **E**, **I**), area 24 (all layers) (**B**, **F**, **J**) and layer V of area 24 (**C**, **G**, **K**) after adjustment by covariates in the left cortical hemisphere (open boxes) and right cortical hemisphere (grey boxes) of patients with schizophrenia (S) and matched controls (C), as well as 95% confidence intervals of adjusted Cohen’s d effect sizes (*d*_adj_) for each outcome (**D**, **H**, **L**). Statistically significant results are indicated (*, *P* < 0.05; **, *P* < 0.01). **CGM**, cortical gray matter.

**
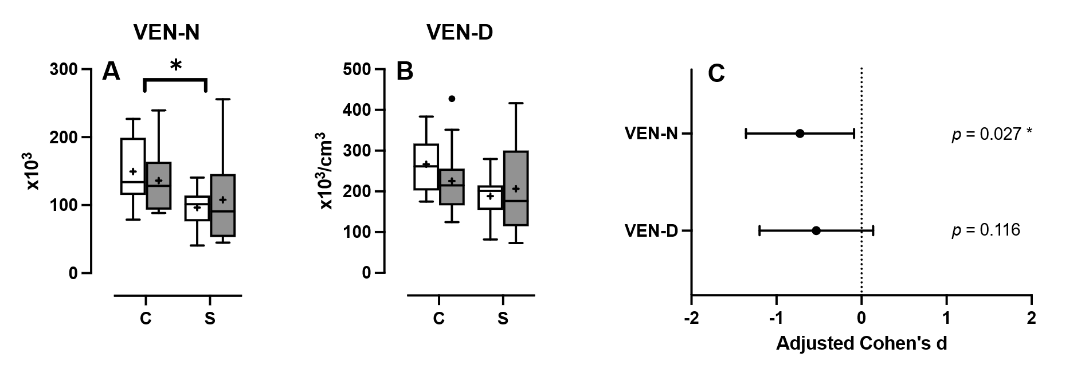
**

**Supplementary Fig. 7: Results of the design-based stereologic analyses after omitting patient S01, Part 2** The panels show Tukey box plots of the estimated total number of VENs (**A**) and the estimated VEN density (**B**) in layer V of area 24 after adjustment by covariates in the left cortical hemisphere (open boxes) and right cortical hemisphere (grey boxes) of patients with schizophrenia (S) and matched controls (C), as well as 95% confidence intervals of adjusted Cohen’s d effect size for each outcome (**C**). Statistically significant results are indicated (*, *P* < 0.05; **, *P* < 0.01). **VEN**, von Economo neuron.

**Supplementary References**

1. Schmitz C. Variation of fractionator estimates and its prediction. *Anat Embryol (Berl)* 1998; **198**(5)**:** 371-397.

2. Schmitz C, Hof PR. Recommendations for straightforward and rigorous methods of counting neurons based on a computer simulation approach. *J Chem Neuroanat* 2000; **20**(1)**:** 93-114.

3. Kreczmanski P, Heinsen H, Mantua V, Woltersdorf F, Masson T, Ulfig N *et al.* Volume, neuron density and total neuron number in five subcortical regions in schizophrenia. *Brain* 2007; **130**(Pt 3)**:** 678-692.
